# Application of an ACE star model–based evidence-based enteral nutrition management protocol in neurocritical care patients

**DOI:** 10.3389/fnut.2026.1786684

**Published:** 2026-03-31

**Authors:** Rong Yuan, Lei Liu, Jiao Mi, Xue Li, Shifang Mao

**Affiliations:** 1 Neurological Intensive Care Unit, Deyang People’s Hospital, Deyang, China; 2 Department of Nursing, Affiliated Hospital of Southwest Medical University, Luzhou, China

**Keywords:** ACE star model, enteral nutrition, evidence-based practice, feeding intolerance, neurocritical care

## Abstract

**Objective:**

To evaluate the efficacy of an evidence-based enteral nutrition management protocol guided by the ACE Star model in neurocritical care patients.

**Methods:**

We collected clinical data from 82 patients admitted to the neurocritical care unit of a tertiary hospital in Sichuan Province. The baseline group comprised 40 patients who met the inclusion and exclusion criteria and were admitted between June and September 2024. The evidence-based practice group included 42 patients meeting the same criteria and admitted between January and April 2025. We compared the two groups with respect to the incidence of feeding intolerance, rates of achieving target feeding goals, changes in serum albumin and prealbumin levels, and healthcare providers’ adherence to standardized enteral nutrition management protocols.

**Results:**

Following implementation of the evidence-based protocol, the incidence of feeding intolerance decreased significantly compared with the baseline period. Improvements were also observed in serum prealbumin and albumin levels and in the proportion of patients achieving target feeding goals. Furthermore, adherence to standardized enteral nutrition practices among healthcare personnel increased significantly after implementation (*p* < 0.05).

**Conclusion:**

A standardized enteral nutrition management protocol based on the ACE Star evidence-based model reduces feeding intolerance, enhances nutritional status, and supports recovery in neurocritical care patients.

## 1 Introduction

Critically ill patients often cannot feed themselves due to gastrointestinal dysfunction, impaired consciousness, or other factors, and may require supplemental nutritional support. Enteral nutrition (EN) is the preferred feeding method for these patients, as it helps prevent atrophy of the gastrointestinal mucosa and enhances neuroendocrine function (1–4). This approach preserves mucosal barrier integrity and immune function in critically ill individuals, reduces infection rates, accelerates wound healing, shortens the duration of invasive mechanical ventilation, decreases hospital length of stay, and lowers mortality (5–7). However, patients with neurological critical illness are particularly susceptible to gastrointestinal dysfunction owing to brain–gut axis dysregulation and trauma-related stress, which impair gastrointestinal motility and delay gastric emptying. Among the associated complications, feeding intolerance (FI) represents the most characteristic clinical manifestation in neurocritically ill patients receiving enteral nutrition (8, 9). Research indicates that the incidence of FI in this population reaches as high as 50%, rising to 83% among those with poor prognosis (10, 11). The development of FI is associated with a 4.6-day increase in hospital stay, an eightfold higher risk of poor prognosis, and a 1.5-fold greater risk of death (12, 13). Clearly, FI directly compromises clinical outcomes in neurocritical care patients, underscoring the urgent need for an evidence-based care protocol to mitigate this complication.

The ACE Star evidence-based model, introduced by American scholar Stevens in 2004, consists of five core steps: problem identification, evidence synthesis, translation assessment, practice integration, and outcome evaluation (14). This systematic framework ensures that clinical decisions are grounded in the best available scientific evidence, thereby maintaining both scientific rigor and practical effectiveness. Nevertheless, research on the application of the ACE Star model to manage FI remains limited. This study therefore aims to clarify the impact of the ACE Star model on feeding intolerance in neurocritical care patients.

## 2 Materials and methods

### 2.1 Study design

This study was conducted in a 24-bed neurocritical care unit (NCU) at a tertiary university-affiliated hospital in China. It included 82 neurocritical care patients admitted to the NCU between June and September 2024 and between January and April 2025. The study received approval from the hospital’s Ethics Committee (ethical approval code: 2023–04-123-K01) and was duly registered in the China Medical Research Registry.

### 2.2 Enrollment

The inclusion and exclusion criteria for this study were as follows.

Inclusion criteria: (1) critically ill patients with neurological disease and existing or potential organ dysfunction; (2) age ≥18 years; (3) initiation of enteral feeding within 48 h of admission; (4) receipt of nasogastric feeding for ≥7 days; and (5) provision of informed consent by the patient or their family members, with willingness to cooperate with the investigators. Exclusion criteria: (1) severe nutritional disorders, digestive insufficiency, or cirrhosis; (2) history of intestinal obstruction; (3) active bleeding from the esophagus, stomach, or intestines; and (4) incomplete clinical data.

### 2.3 Methods

This study employed a historical control design. The conventional care group consisted of 40 patients admitted to the neurocritical care unit between June and September 2024 who met the inclusion and exclusion criteria. The evidence-based practice group included 42 patients admitted between January and April 2025 who met the same criteria.

Conventional Care Group: Physicians, nurses, dietitians, and physiotherapists delivered interventions independently within their respective disciplines, communicating only when necessary. Feeding routes were selected based on clinical experience, and feeding rates were set and adjusted accordingly. Medications to promote gastrointestinal motility were administered as prescribed.

Evidence-Based Practice Group: A standardized enteral nutrition management protocol based on the ACE Star evidence-based model was implemented. The research followed five steps: problem establishment, evidence synthesis, translation into clinical recommendations, practice integration, and effect evaluation. Each element is detailed as follows.

### 2.4 Establishment of the problem

Through clinical scenario analysis, the evidence-based question was determined based on current clinical practice and needs: What measures can reduce the incidence of FI? Following the PIPOST framework, the target population (P) is neurocritical care patients; the intervention (I) comprises assessment, intervention, and management of FI; professionals applying the evidence (P) are healthcare personnel in the neurocritical care unit; the outcome (O) is the incidence of enteral FI in neurocritical care patients; the setting (S) is the neurocritical care unit; and the type of evidence (T) includes clinical practice guidelines, systematic reviews, evidence summaries, and expert consensus.

### 2.5 Synthesis of evidence

This project employs a top-down retrieval strategy based on the ‘6S’ evidence pyramid principle. The Chinese search keywords include terms such as ‘neurocritical care’, ‘neurological critical care’, ‘neurosurgery critical care’, ‘ischemic stroke’, ‘hemorrhagic stroke’, ‘stroke’, ‘cerebral infarction’, ‘cerebrovascular disease’, ‘severe traumatic brain injury’, ‘intracerebral hemorrhage’, ‘feeding tolerance’, ‘feeding intolerance’, ‘protocol’, ‘process’, ‘management’, ‘guideline’, ‘consensus’, ‘summary of best evidence’, ‘systematic review’, ‘meta-analysis’, and ‘clinical decision-making’. English search terms are: ‘neurological intensive’, ‘NCU’, ‘NICU’, ‘neurological severe disease’, ‘neurological intensive care unit’, ‘FI’, ‘feeding intolerance’, ‘diarrhea’, ‘vomiting’, ‘gastric residual volume’, ‘project’, ‘guidance’, ‘meta-analysis’, ‘systematic review’, ‘consensus’, and ‘system review’.

The search period spanned from database inception to July 2024, including Chinese and English guideline portals and relevant professional association websites such as UpToDate, BMJ Best Practice, the Australian Joanna Briggs Institute (JBI), the Center for Evidence-Based Healthcare, Guidelines International Network (GIN), National Institute for Health and Care Excellence (NICE), Scottish Intercollegiate Guidelines Network (SIGN), MedlinePlus, China National Knowledge Infrastructure (CNKI), VIP Chinese Data, Wanfang Database, CBM, PubMed, Medline, Cochrane Library, and Web of Science.

Inclusion criteria were: (1) adult patients (≥18 years) with neurological critical illness; (2) studies involving assessment, screening, or nursing interventions for feeding intolerance; and (3) study types including clinical decision aids, practice guidelines, evidence summaries, systematic reviews, or expert consensus statements. Exclusion criteria included: (1) incomplete or brief guidelines containing only introductions, tables of contents, abstracts, etc.; (2) literature with incomplete information or unavailable full texts; (3) duplicate publications; and (4) literature of insufficient quality.

Ultimately, 15 studies were included in this synthesis: three guidelines, two evidence summaries, three expert consensus documents, and seven systematic reviews.

### 2.6 Translation into clinical recommendations

Two research nurses, both holding master’s degrees and trained at Fudan University’s Evidence-Based Nursing Center, evaluated the primary evidence sources within the evidence summaries using the Appraisal of Guidelines for Research & Evaluation II (AGREE II) instrument (15), the 2016 JBI Critical Appraisal Checklist for Text and Opinion Papers (including expert consensus articles) (16), and the A Measurement Tool to Assess Systematic Reviews (AMSTAR). When discrepancies arose in evaluation results, a third-party arbitrator was consulted, resulting in a final set of 22 optimal summarized evidence items. A multidisciplinary research team, including one neurocritical care medical director, one neurocritical care head nurse, one nutritionist, one rehabilitation therapist, three critical care specialist nurses (one of whom holds critical care ultrasound certification), and two research nurses, then assessed the 30 extracted evidence items against the FAME criteria: feasibility, appropriateness, clinical meaningfulness, and effectiveness. The team applied the 2014 Evidence Grading Recommendation System (15), in which Level 1 denotes the highest quality and Level 5 the lowest. Evidence reliability was determined based on study design, methodological rigor, and statistical approach, yielding a five-tier grading classification. These graded items were subsequently translated into 10 review criteria, detailed in Table 1.

**Table 1:** Evidence content and review criteria for standardized management of enteral nutrition in neurocritical care patients.

Item	Evidence content	Review criteria
Nutritional status assessment	1. The establishment of a three-dimensional indicator system encompassing structure, process, and outcome, in conjunction with the formation of a multidisciplinary team for nutritional management, is recommended. The composition of the team should include specialists in neurocritical care, specialist nurses, dietitians, and rehabilitation therapists (19, 29)	
	2. The Nutrition Risk Screening 2002 (NRS-2002) is recommended for the assessment of nutritional risk in stroke patients. Scores of 3 or higher indicate a requirement for enteral nutrition (EN) support within 48 h, while scores below 3 should be reassessed after 1 week. In instances where precise BMI measurements are not feasible for critically ill patients, the Nutrition Risk in Critical Illness (NUTRIC) score is a recommended alternative (19, 30–32)	1. It is incumbent upon nurses to conduct nutritional risk screening using the NRS-2002 within 4 h of a patient’s admission to the ward.
Feeding method	3. It is recommended that the route of enteral nutrition be optimized based on factors such as the patient’s nutritional risk, swallowing ability, level of consciousness, anticipated duration, and risk of complications (19)	2. Nurses may select feeding routes in accordance with standardized procedures for enteral nutrition pathways.
	4. For patients with feeding intolerance and high aspiration risk, or those exhibiting gastric residual volume (GRV) exceeding 100 mL, it is recommended to reduce infusion rates or administer prokinetic agents. In the absence of improvement within 24 to 48 h, post-pyloric feeding is recommended, with nasojejunal tube placement for enteral nutritional support. Once clinical stability has been achieved, with GRV consistently below 100 mL for a period of 1 week and normal bowel sounds, the gradual discontinuation of the nasojejunal tube may be contemplated (18, 21, 29–31, 33, 34)	
Feeding temperature	5. It has been demonstrated that elderly patients demonstrate improved tolerance to warmed enteral nutrition solutions, particularly those experiencing feeding intolerance such as constipation or diarrhea. It is recommended that enteral nutrition solutions be administered at a temperature of 38 °C to 42 °C (18, 19, 21, 31, 32)	3. Warm enteral feeding solutions are administered using an applicator heater for infusion.
Feeding rate	6. The recommendation for acute-phase and severely unwell patients is that they should receive continuous enteral nutrition (19, 35)	4. The initial enteral feeding rate is selected on the basis of monitoring data pertaining to the motility of the stomach or intestines.
	7. During enteral feeding, it is recommended to commence nasogastric feeding at a rate of 15–50 mL/h, increasing by 10–50 mL/h every 4–24 h over a period of 6 days, followed by gradual escalation to the target feeding rate (18)	
	8. In the case of patients exhibiting feeding intolerance, the utilization of trophic feeding for a period of 6 days may be contemplated (18, 31)	
Feeding position	9. The recommendation is that the head of the bed should be elevated to a range of 30°–45° when enteral feeding is being administered via a gastric tube (18, 21, 30–32)	5. For patients receiving enteral nutrition without specific requirements for head elevation, the head of the bed should be raised to 30–45 degrees.
	10. It is recommended that the patient be maintained in the original position for a minimum of 30 min following enteral nutrition, with the exception of circumstances such as low intracranial pressure or following lumbar puncture (18, 19, 21)	6. The maintenance of this original position for a period of at least 30 min is to be ensured following the ingestion of enteral feeding.
Identification of Feeding Intolerance	11. Patients receiving nasogastric feeding should undergo daily assessment of their tolerance to enteral nutrition. This should include a physical examination and an evaluation of any symptoms of intolerance, such as diarrhea or constipation (18, 21)	7. The responsible nurse shall conduct daily physical examinations and assess patients’ enteral nutrition tolerance using ultrasound in conjunction with the Enteral Nutrition Tolerance Score, recording the findings accordingly.
	12. Routine monitoring of GRV is not recommended unless there is a high risk of aspiration or intolerance (19, 21, 36, 37)	
	13. For critically ill patients with gastrointestinal intolerance or a high risk of aspiration undergoing enteral feeding, the gastric residual volume (GRV) should be monitored every 4 h. If the GRV exceeds 250 mL, post-pyloric feeding, promotion of gastrointestinal motility, and elevation of the head of the bed are recommended (18, 21, 31)	
	14. The syringe aspiration method is recommended for assessing gastric residual volume in patients. Ultrasound monitoring may be used where available (18, 19)	
Management of feeding intolerance	15. In addition to reducing the feeding volume or slowing the infusion rate when diarrhea occurs, one may also alter the method of administering the nutritional solution, adjust its formulation, or warm the solution prior to administration (29–31)	8. It is imperative that management is conducted in accordance with the standardized protocol for complications arising from enteral nutrition.
	16. If diarrhea occurs, nasogastric feeding should not be stopped straight away. Instead, the feeding rate should be reduced and/or the total volume of nutritional solution decreased while the underlying cause is identified to determine the appropriate treatment approach (21, 31)	
	17. In cases of constipation, it is recommended that fluids be replenished promptly, and that nutritional supplements containing mixed dietary fiber or probiotics be administered for enteral nutrition (EN). In addition, prokinetic agents should be used to intervene, and concurrent abdominal massage is recommended to facilitate bowel movements (21, 30, 32)	
	18. In instances of vomiting and abdominal distension, it is recommended that the infusion rate be reduced, and, if necessary, the total infusion volume be decreased, while conducting a thorough investigation into the underlying cause and administering treatment aimed at mitigating the symptoms. In the event of persistent symptoms, the transition to parenteral nutrition is recommended (30)	
	19. In the event of two consecutive measurements of gastric residual volume exceeding 250 mL, the utilization of prokinetic agents is recommended (18)	
Enhance gastrointestinal tolerance	20. For patients undergoing enteral nutrition requiring suctioning, it is recommended to implement measures such as immediately ceasing feeding, performing superficial suctioning, employing positional management, and minimizing irritation to reduce the incidence of aspiration and reflux (19)	9. For patients receiving enteral nutrition, the shallow suctioning technique should be employed.
	21. The utilization of probiotics is recommended, as they may serve to reduce the incidence of diarrhea, vomiting and constipation (21)	
	22. It is recommended that patients receive treatment to improve their gastrointestinal function. This is achieved through the application of abdominal massage, abdominal heat application, and early bed rest combined with passive exercises (19)	10. Help rehabilitation therapists conduct early bedside rehabilitation exercises with patients whose vital signs are stable.

### 2.7 Practice integration

#### 2.7.1 Review of the current situation

In the Evidence-Based Practice Group, the Director of Neurocritical Care oversees the overall planning of evidence-based practice initiatives; the Head Nurse provides clinical consultation and coordinates implementation; one Nutritionist reviews the development and quality control of indicators; one Rehabilitation Therapist and three Critical Care Specialist Nurses are responsible for implementing the evidence; and two Research Nurses manage evidence synthesis, deliver research protocol training, and conduct data collection and analysis. (1) Direct observation method: Indicators 2, 3, 4, 5, 6, 8, 9, and 10 were recorded through direct observation of nursing staff by members of the Evidence-Based Practice Team. (2) Document review method: Indicators 1 and 7 were obtained by reviewing nursing records and medical orders.

#### 2.7.2 Analysis of barriers

Barriers were identified through expert panel interviews and included the following: (1) insufficient knowledge among neurocritical care personnel regarding enteral nutrition; (2) absence of departmental guidelines for selecting appropriate feeding routes; (3) inadequate emphasis by healthcare staff on preventing enteral FI; (4) lack of standardized assessment tools within the department for evaluating enteral nutrition tolerance; and (5) insufficient use of ultrasound assessments to detect enteral FI.

#### 2.7.3 Implementing change initiatives

**Figure 1:**
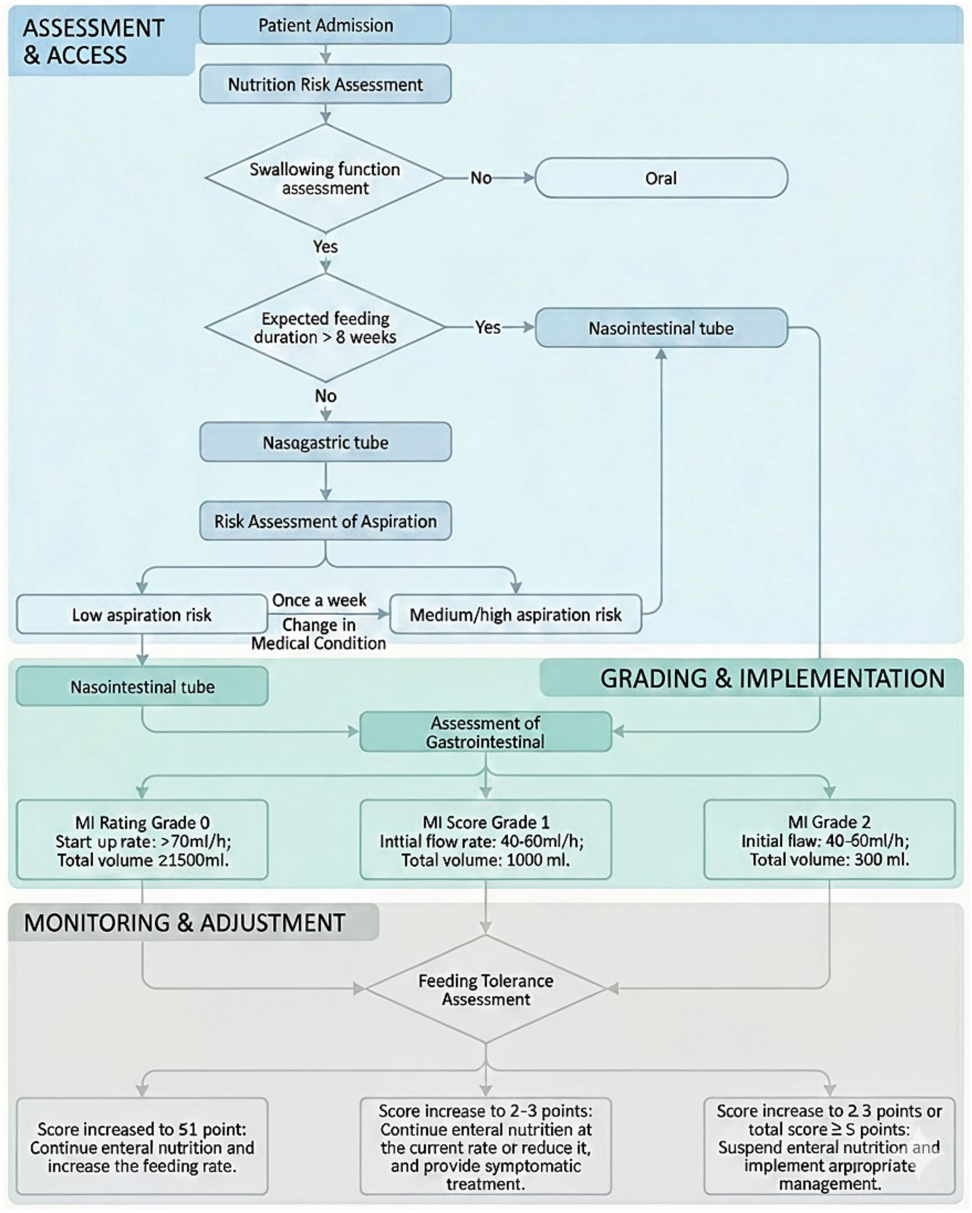
Standardized enteral feeding protocol for neurocritical care patients.

Based on the barrier analysis, hospital resources were consolidated to develop evidence-based quality management strategies and implementation protocols. These include the Standardized Enteral Nutrition Feeding Protocol for Neurocritical Care Patients (Figure 1) and the Management Protocol for Enteral Feeding Intolerance Complications in Neurocritical Care Patients. Upon admission, integrated medical and nursing teams assess nutritional risk and select enteral feeding routes according to aspiration risk stratification. Ultrasound is used to evaluate gastrointestinal motility, and the gastric motility index (MI) (17) is calculated to guide adjustments in the initial enteral nutrition infusion rate. During feeding, daily bedside assessments are performed to monitor feeding tolerance, and enteral feeding rates are modified accordingly. If complications related to feeding intolerance occur, standardized protocol-driven interventions are promptly initiated. Further details are provided in Figure 1.

Ultrasound Team Training Program: Specialist nurses certified in critical care ultrasound delivered focused training sessions held every 2 weeks. The curriculum covered foundational principles of critical care ultrasound, operation and maintenance of ultrasound equipment, upper gastrointestinal anatomy and physiology, gastric antrum measurement techniques, ultrasound-guided esophageal tube confirmation, ultrasound-assisted nasoenteric tube placement, and monitoring of gastric residual volume. Instruction employed a workshop-based format supplemented with multimedia resources, including teaching aids, annotated images, and procedural videos. Practical competency assessments were administered at the conclusion of each session.

Departmental Medical and Nursing Knowledge and Skills Training: Guided by the 10 review criteria, comprehensive training was provided to all medical and nursing staff in the department. The neurocritical care director presented on the definition, high-risk factors, and clinical management of enteral feeding intolerance in neurocritical care patients. A nutritionist delivered a lecture on selecting appropriate enteral nutritional formulations. Two critical care specialist nurses demonstrated the use of the enteral feeding intolerance risk assessment form and the modified Wada water swallow test. The Rehabilitation Therapy Department addressed assessment methods, stepwise progression, and monitoring strategies for early graded mobilization activities. Written post-training assessments were collected immediately after the session. Any questions with an error rate exceeding 50% were reviewed during morning handover meetings or departmental conferences to ensure all staff achieved proficiency in enteral nutrition–related feeding intolerance prevention knowledge and procedures.

### 2.8 Effect evaluation

(1) Incidence of feeding intolerance, compared between the two groups. In this study, the criteria for determining FI were as follows: vomiting or reflux, defined as the passage of gastric contents through the esophagus and out of the mouth, was identified by the presence of nutrient fluid in the mouth or spillage from a nasogastric or nasoenteric tube upon opening; diarrhea was defined as more than three bowel movements per day with unformed stools containing at least 80% water; constipation was defined as no bowel movement for three consecutive days or one bowel movement every 2 to 3 days with dry, hard stools weighing less than 50 g; aspiration referred to the entry of gastric contents into the airway; high gastric residual volume was defined as a daily volume of 500 mL or more; and gastrointestinal bleeding was diagnosed when patients vomited or regurgitated bloody fluid, had visible blood in the stool accompanied by a positive occult blood test, or received physician confirmation (e.g., via gastroscopy). The presence of one or more of these criteria indicated FI. (2) Nutrition management–related indicators in both groups before and after evidence-based practice included the target feeding achievement rate, changes in serum albumin levels, and changes in serum prealbumin levels. (3) A self-developed questionnaire was used to assess changes in healthcare professionals’ knowledge levels before and after evidence-based practice, as well as the implementation rate of standardized enteral nutrition management measures.

### 2.9 Statistical analysis

Quantitative data that followed a normal distribution were expressed as mean ± standard deviation (x̄±s). Intergroup comparisons were performed using the independent-samples t-test. Qualitative data were presented as frequencies and percentages, and intergroup comparisons were conducted using the chi-square test. A *p*-value < 0.05 was considered statistically significant.

Preliminary experiments yielded a calculated required sample size of 29 participants per group, for a total of 58 participants across both groups. Accounting for an anticipated 10% attrition rate, the sample size was adjusted to 29 / (1–0.10), resulting in approximately 32 participants per group. This adjustment established a minimum required total sample size of 64 participants. The study ultimately enrolled 82 patients (40 in the control group and 42 in the intervention group), which satisfies the stipulated sample size requirement.

## 3 Results

### 3.1 Patient characteristics

This study included 82 patients (47 males and 35 females) with a mean age of 65.66 ± 15.03 years. There were no significant differences in baseline characteristics between the two groups (*p* > 0.05), as detailed in Table 2.

**Table 2:** Comparing general patient characteristics between two groups.

Group	Number of examples	Sex (cases)		Age (years)	BMI (cases)	GCS scores (points)	NRS 2002 score (points)	Route of feeding (cases)	
		Male	Female					Nasogastric tube	Nasointestinal tube
Conventional care group	40	22	18	65.15 ± 14.05	22.49 ± 4.11	8.15 ± 2.94	3.43 ± 0.50	32	8
Evidence-based practice group	42	25	17	66.14 ± 16.07	23.60 ± 2.89	8.52 ± 2.96	3.55 ± 0.50	31	11
*χ* ^2^		0.171		−0.297^1^	−1.423^1^	0.574^1^	−1.105^1^	0.441	
*p*		0.679		0.767	0.159	0.568	0.272	0.507	

1 t-value.

### 3.2 Comparison of complication rates between the two groups

As shown in Table 3, the incidence of FI decreased after implementation of the evidence-based practice compared with the pre-implementation period, and this difference was statistically significant (*p* < 0.05). Furthermore, multivariate logistic regression analysis was performed (see Table 4). After adjusting for potential confounding variables, group assignment was strongly associated with FI (OR = 0.156, 95% CI: 0.051–0.479, *p* = 0.001).

**Table 3:** A comparison of the incidence of fi in two groups of patients receiving enteral nutrition.

Group	Number of examples	FI	Diarrhea	Constipation	Vomiting/regurgitation	Aspiration	High gastric residue	Gastrointestinal bleeding
Conventional care group	40	28	15(37.5)	12(30.0)	8(20.0)	9(22.5)	10(25.0)	11(27.5)
Evidence-based practice group	42	13	4(9.52)	4(9.52)	3(7.14)	1(2.34)	1(2.38)	3(7.14)
*χ* ^2^		12.495	9.008	5.470	2.916	5.980	9.024	5.997
*p*		<0.001	0.003	0.019	0.088	0.014	0.003	0.014

**Table 4:** Logistic regression analysis of risk factors for developing FI in neurocritical care patients.

Independent variable	*B*	*SE*	*Wald*	*p*	*OR* (95% CI)
Grouping	−1.859	0.573	10.539	0.001	0.156(0.051–0.479)
Age	−0.008	0.029	0.084	0.772	0.992(0.938–1.049)
Sex	0.039	0.547	0.005	0.943	1.040(0.356–3.04)
BMI	0.050	0.094	0.286	0.593	1.051(0.875–1.263)
GCS layering	0.000	0.095	0.000	0.998	1.000(0.829–1.205)
NRS2002	−0.440	0.939	0.219	0.640	0.644(0.102–4.058)
Route of feeding	2.400	0.758	10.024	0.002	11.025(2.495–48.713)
Constant	−0.613	3.449	0.032	0.859	0.542

### 3.3 Comparison of two sets of nutrition-related indicators

Compared with pre–evidence-based practice levels, serum prealbumin and serum albumin concentrations, as well as the target feeding achievement rate, increased significantly (*p* < 0.05), as shown in Table 5.

**Table 5:** Comparison of nutrition-related indicators between two groups of patients.

Group	Number of examples	Achieving target feeding standards	Serum albumin variation value	Serum prealbumin variation value
Conventional care group	40	15	1.187 ± 0.79	3.812 ± 1.47
Evidence-based practice group	42	29	3.96 ± 2.51	5.767 ± 2.81
*χ* ^2^		9.899	−6.809^1^	−3.971^1^
*p*		0.002	<0.001	<0.001

1 t-value.

### 3.4 Comparison of healthcare professionals’ knowledge levels regarding enteral nutrition management before and after evidence-based practice

Before the evidence-based practice initiative, the awareness rate of relevant evidence among neurocritical care personnel was 68.89%. Following implementation, this rate increased to 90.58%.

### 3.5 Comparison of pre- and post-intervention review indicators in evidence-based practice

The standardized implementation rate of enteral nutrition among healthcare personnel increased significantly compared with pre–evidence-based practice levels (*p* < 0.05), as shown in Table 6.

**Table 6:** A comparison of healthcare professionals’ review indicators for preventing enteral nutrition-related FI before and after the implementation of evidence-based practice.

Review criteria	Conventional care group(*n* = 52)			Evidence-based practice group(*n* = 52)			*p*
	Implemented (cases)	Not implemented (cases)	Implementation rate (%)	Implemented (cases)	Not implemented (cases)	Implementation rate (%)	
Indicator 1	48	4	92.31%	52	0	100.00%	0.041
Indicator 2	15	37	28.85%	48	4	92.31%	<0.001
Indicator 3	45	7	86.54%	51	1	98.08%	0.027
Indicator 4	10	42	19.23%	39	13	75.00%	<0.001
Indicator 5	45	7	86.54%	52	0	100.00%	0.006
Indicator 6	43	9	82.69%	52	0	100.00%	0.002
Indicator 7	7	45	13.46%	49	3	94.23%	<0.001
Indicator 8	0	52	0.00%	50	2	96.15%	<0.001
Indicator 9	44	8	84.62%	51	1	98.08%	0.015
Indicator 10	46	6	88.46%	52	0	100.00%	0.012

## 4 Discussion

The present study implemented the ACE Star evidence-based management model (2, 14), which uses a five-step framework to establish a standardized enteral nutrition care pathway aimed at improving patient outcomes. Our findings indicate that systematic implementation of evidence-based practice was associated with markedly improved tolerance of enteral nutrition. Specifically, after adopting this approach, the risk of FI was effectively reduced, and patients showed concurrent improvements in nutritional status, reflected by increased serum prealbumin and albumin levels, and a higher rate of achieving target feeding goals. At the same time, adherence to standardized enteral nutrition protocols among healthcare professionals increased substantially. Together, these interrelated improvements suggest that our evidence-based practice framework may foster a virtuous cycle, translating standardized clinical practices into enhanced patient outcomes, a dynamic with considerable potential for broader clinical application.

Feeding intolerance is one of the most common complications of enteral nutrition therapy and can undermine feeding efficacy while increasing the risk of adverse events. Multiple factors contribute to FI, and its prevention remains a central focus in clinical practice (18). Before implementing evidence-based practice, the lack of standardized protocols led to suboptimal FI prevention outcomes. In this study, the incidence of FI was significantly lower in the evidence-based practice group than in the routine care group (*p* < 0.05). After adjusting for potential confounders, including age, Glasgow Coma Scale (GCS) score, nutritional risk score, and feeding route, multivariable logistic regression analysis showed that the evidence-based practice intervention was independently associated with a reduced risk of FI (OR = 0.156, 95% CI: 0.051–0.479, *p* = 0.001). This reduction likely stems from multiple preventive strategies embedded in the evidence-based protocols, such as using scientifically grounded feeding pathways that guide selection of feeding routes based on swallowing function, aspiration risk, and overall clinical condition. Bedside ultrasound-guided tube placement may further support timely initiation of enteral nutrition (19, 20). Additionally, daily bedside assessments combined with ultrasound evaluation of gastrointestinal motility enable prompt adjustments to feeding rates to meet individual nutritional needs. Should complications arise, protocol-driven interventions for managing enteral nutrition–related complications (18, 21) may collectively reduce gastrointestinal burden and improve feeding tolerance.

Nutritional management is a critical component of treatment for neurocritical care patients. Patients with acute neurological illness are often at high risk of malnutrition due to impaired consciousness, dysphagia, and vagal inhibition (22, 23). FI can further exacerbate this risk and increase the likelihood of adverse outcomes, including prolonged invasive mechanical ventilation, extended hospital stays, and higher rates of infection and mortality (12, 13). Moreover, complications associated with specific manifestations of FI—such as pulmonary infections from reflux or aspiration, incontinence-associated dermatitis due to diarrhea, and pressure injuries resulting from malnutrition (24), may further worsen patient outcomes. The present study found that the evidence-based practice group achieved significantly higher target feeding achievement rates and showed greater improvements in serum albumin and prealbumin levels compared with the control group (*p* < 0.05). This improved tolerance enabled higher delivery of prescribed enteral nutrition, ensuring adequate energy and protein intake. The concurrent increases in serum prealbumin and albumin provide direct biological evidence of sustained nutritional improvement. Prealbumin, a sensitive short-term nutritional marker, rises in response to enhanced protein synthesis. Similarly, rising albumin levels may reflect favorable trends in long-term nutritional status and clinical prognosis (7, 25, 26), suggesting a potential pathway linking improved feeding tolerance to better nutritional outcomes.

This study also observed an increase in the standardized implementation rate of enteral nutrition among healthcare professionals. Historically, enteral nutrition management has relied heavily on individual experience and professional judgment (27). The evidence-based approach described here may have facilitated the translation of current evidence into actionable clinical standards (28) through standardized operating procedures, systematic training programs, and ongoing quality feedback. This process not only improved clinicians’ understanding of key parameters such as feeding rate, formula concentration, and temperature, but more importantly, may have helped cultivate an evidence-based, patient-safety–centered culture of quality care.

## 5 Limitations

This study had several limitations. First, the clinical setting imposed practical constraints that limited the sample size. Second, the review indicators were adapted to the specific clinical context and available resources at this institution; therefore, their applicability may vary across hospitals of different tiers. Finally, the study did not assess long-term clinical outcomes such as ICU length of stay or mortality. Future research will aim to more comprehensively evaluate the overall impact of this intervention.

## 6 Conclusion

This evidence-based practice initiative, grounded in the ACE Star model, successfully standardized enteral nutrition care for neurocritical care patients. The intervention was associated with a reduced incidence of FI, improved nutrition-related indicators, and enhanced adherence by healthcare professionals to enteral nutrition implementation protocols.

## Data Availability

The original contributions presented in the study are included in the article/supplementary material, further inquiries can be directed to the corresponding author.
